# Specific resistance prevents the evolution of general resistance and facilitates disease emergence

**DOI:** 10.1111/jeb.14170

**Published:** 2023-03-27

**Authors:** Samuel V. Hulse, Janis Antonovics, Michael E. Hood, Emily L. Bruns

**Affiliations:** 1 University of Maryland at College Park, College Park, Maryland, USA; 2 University of Virginia, Charlottesville, Virginia, USA; 3 Amherst College, Amherst, Massachusetts, USA

**Keywords:** evolutionary feedback, general resistance, host shift, quantitative resistance, specific resistance, spillover

## Abstract

Host-shifts, where pathogens jump from an ancestral host to a novel host, can be facilitated or impeded by standing variation in disease resistance, but only if resistance provides broad-spectrum general resistance against multiple pathogen species. Host resistance comes in many forms and includes both general resistance, as well as specific resistance, which may only be effective against a single pathogen species or even genotype. However, most evolutionary models consider only one of these forms of resistance, and we have less understanding of how these two forms of resistance evolve in tandem. Here, we develop a model that allows for the joint evolution of specific and general resistance and asks if the evolution of specific resistance drives a decrease in the evolution of general resistance. We also explore how these evolutionary outcomes affect the risk of foreign pathogen invasion and persistence. We show that in the presence of a single endemic pathogen, the two forms of resistance are strongly exclusionary. Critically, we find that specific resistance polymorphisms can prevent the evolution of general resistance, facilitating the invasion of foreign pathogens. We also show that specific resistance polymorphisms are a necessary condition for the successful establishment of foreign pathogens following invasion, as they prevent the exclusion of the foreign pathogen by the more transmissible endemic pathogen. Our results demonstrate the importance of considering the joint evolution of multiple forms of resistance when evaluating a population's susceptibility to foreign pathogens.

## 1 ∣ INTRODUCTION

Understanding the causes and risks of pathogen host-shifts remains one of the most important challenges in disease ecology (Borremans et al., 2019). Host-shifts occur when a pathogen is spread from an ancestral host to a new host species in which it shares no coevolutionary history. The importance of host-shift pathogens is clear for their hosts: a meta-analysis examining the evolutionary history of host–pathogen associations across all taxa found evidence of historic host-shifts in 93% of studies (Vienne et al., 2013). While much of the focus of host-shift risk has been on the phylogenetic distance between hosts (Parker et al., 2017; Streicker et al., 2010), within-species genetic variation in resistance may also play a crucial role (Lerner et al., 2021). Hosts utilize a diverse array of mechanisms that reduce the likelihood of infection, which vary in their effectiveness against a potential host-shift pathogen. Therefore, the maintenance and frequency of particular forms of resistance may be vital in preventing host-shifts.

Many host defence systems include both general (protection against a broad array of pathogen genotypes and species) and specific resistance mechanisms (protection against a single pathogen species or genotype). For example, in vertebrates the inflammatory response is a general resistance response against a wide range of potentially pathogenic microbes (Sorci & Faivre, 2009), while the humoral response is calibrated to specific pathogens (Pier et al., 2004). In plants, general resistance can arise from both constitutive structural defences such as waxy cuticles (Chassot & Métraux, 2005) and induced responses triggered by general microbe-associated molecules like flagellin and chitin (Gómez-Gómez & Boller, 2002; Thompson & Burdon, 1992), while specific resistance can be triggered by R-gene recognition of high species- and genotype-specific secreted pathogen effectors (Jones & Dangl, 2006; Ngou et al., 2022).

The degree of resistance specificity may influence the host's protection against foreign pathogens. General resistance, in the form of positive correlations in resistance to multiple pathogens has been detected in several host–pathogen systems (Chung et al., 2012; Fellowes et al., 1999). Recently, Lerner et al. (2021) showed a strong positive correlation between family-level resistance to an endemic fungal pathogen and a closely related ‘foreign’ pathogen that causes occasional cross-species disease transmission in the herbaceous plant *Silene vulgaris*. They further showed, using a deterministic model that assumed pleiotropy in resistance to an endemic and foreign pathogen, that the sign and magnitude of this pleiotropy affected foreign pathogen invasion, even when the average resistance remained constant. However, their analysis did not investigate the evolutionary mechanisms producing correlations in resistance to endemic and foreign pathogens.

Over the past few decades, a rich body of literature has emerged to study the evolutionary dynamics of specific resistance controlled by single resistance genes (Tellier & Brown, 2007) and general resistance with continuous variation (Boots & Haraguchi, 1999). However few studies have considered their evolution jointly (Frank, 2000), even though evolutionary outcomes are typically different when multiple interrelated traits are allowed to evolve simultaneously (Restif & Koella, 2004). Using a Lotka-Volterra-based model, Frank (2000) found a negative correlation between host investment in general and specific resistance: hosts tended to invest in either form of resistance, but not both. However, the consequences of these evolutionary dynamics for foreign pathogen invasion have not been investigated. Moreover, the initial invasion by a foreign pathogen is just the first step in a host-shift event. To successfully establish within a new host population, pathogens must overcome not only host resistance structure but also escape exclusion by the resident endemic pathogens.

Here, we develop a compartmental Susceptible-Infected (SI) model of a host resistance which includes both general and specific resistance. We examine the evolutionary outcomes for both forms of resistance in (1) the presence of a single endemic pathogen and (2) the presence of both an endemic pathogen and a less infectious foreign pathogen. Our model assumes a sterilizing pathogen with frequency-dependent transmission, such as the anther-smut plant disease system (Bernasconi et al., 2009). In plants, specific resistance is typically a qualitative trait (Jones & Dangl, 2006), and resistance polymorphism can be maintained through moderate costs, providing there is also overall population regulation (Antonovics & Thrall, 1994). In contrast, general resistance is usually a continuous polygenic trait (Poland et al., 2009), with polymorphism depending on the resistance-costs tradeoff curvature (Boots & Haraguchi, 1999). To account for these genetic differences, we assume that specific resistance evolves through frequency changes of two alleles, S and R, which vary in their resistance to an endemic pathogen but do not affect the foreign pathogen. General resistance is assumed to evolve as a continuous trait conferring resistance to both endemic and foreign pathogens. First, we take a reductionist approach, and examine the case where the evolution of both general and specific resistance occurs with a single, endemic pathogen and ask how these outcomes affect the invasion potential of foreign pathogens. We then expand our model to include a foreign pathogen and examine the conditions in which a foreign pathogen can invade and persist, and how this feeds back on the genetic structure of their hosts.

## 2 ∣ MODEL DESCRIPTION

### 2.1 ∣ Overview

Our model is loosely based on the anther-smut disease of *S. vulgaris.* Anther smut is caused by a fungal pathogen (*Microbotryum silenes-inflatae*) which sterilizes infected plants by replacing pollen production with spore production and aborting the ovary. Infection is generally permanent, with no recovery, and results in complete sterility without impacting mortality (Bruns et al., 2017). The pathogen is spread via pollinators, and infection is expected to be frequency dependent (Thrall et al., 1995). Within the system, both specific and general resistance have been observed (Chung et al., 2012; Lerner et al., 2021), as well as host-shifts, both historical (Refrégier et al., 2008) and contemporary (Antonovics et al., 2002).

Here, we develop a deterministic SI model that allows host evolution to occur independently at two resistance loci: one for specific resistance, modelled as a discrete trait, and the other for general resistance, modelled as a continuous trait. With this model, we first examine the evolutionary outcomes with a single endemic pathogen, and then with both an endemic pathogen and a ‘foreign’ host-shift pathogen. Both pathogen types are modelled after the dynamics of anther-smut disease. We assume that both hosts and pathogens are haploid with no recombination (this latter assumption is later relaxed), that transmission is frequency dependent (density-dependent transmission was also analysed, see Data S1; Figure S1 and S2), and that infection results in sterilization but does not affect host mortality.

### 2.2 ∣ Disease transmission

In our model, the numbers of individuals infected by the two pathogen species are given by $I_{e}$, an endemic pathogen and $I_{f}$, a foreign pathogen, with base transmission parameters $\beta_{e}$ and $\beta_{f}$. We assume that the baseline transmission rate of the endemic pathogen $\beta_{e}$ is greater than the baseline transmission rate for the foreign $\beta_{f}(\beta_{e}>\beta_{f})$ since newly host-shifted foreign pathogens likely have not had time to adapt to their new hosts, and evolve higher transmission rates (Longdon et al., 2014). Previous work has demonstrated a reduced transmission rate for foreign *Microbotryum* species shifting to new hosts (Lerner et al., 2021) and phylogenetically related fungal pathogens more broadly (Bettgenhaeuser et al., 2014; Lee et al., 2017; Schweizer, 2007).

### 2.3 ∣ Host resistance

Hosts can reduce pathogen transmission rates through both specific resistance (with binary variation) and general resistance (with incremental variation). Specific resistance is determined by a single resistance gene with two alleles, S and R. We assume that the S allele confers no specific resistance while the R allele reduces the transmission rate of the endemic pathogen by a factor of r, but provides no defence against the foreign pathogen, akin to a host resistance gene which evolved to detect a highly specific pathogen effector molecule unique to the endemic pathogen. In uninfected hosts, we assume that specific resistance comes at a cost relative to susceptibility, and reduces host fecundity by a fixed factor $c_{r}$.

General resistance is determined by a single gene with many alleles, $\{q_{i}{\}}_{i\leq n}$ that reduces the transmission rate of both endemic and foreign pathogens by a factor of q. This represents a scenario where a constitutive, continuously variable trait like cuticle thickness reduces the transmission rates of all pathogens. We assume a fecundity cost of general resistance $c_{q}$, specified by a power function:

$$c_{q_{{}_{i}}}=1-(1-q_{{}_{i}}{)}^{\theta }$$

where the parameter θ defines the shape of the resistance-cost tradeoff curve. When θ<1, the costs of resistance are accelerating while θ>1, the costs are decelerating (see Boots & Haraguchi, 1999).

We allow q to evolve through a deterministic adaptive dynamics approach (Boots & Haraguchi, 1999; Metz et al., 1995). This approach can be contrasted with optimization-based models, which do not include game-theoretic effects, and may produce different outcomes (Data S2, Figure S3). At each iteration, small mutations of the focal trait are introduced to determine whether new mutations can invade the established population. After the introduction of new mutations, the allele frequencies are allowed to equilibrate, after which a new round of mutations is introduced. This approach assumes that allele frequency changes are rapid relative to the introduction of new mutations.

Within an individual host, we assume that the two forms of resistance are multiplicative, as would be the case for an individual that carries a specific resistance gene and has evolved a thicker cuticle. This also prevents the total resistance level from being >1. We assume that the costs of general and specific resistance are additive. This simplifying assumption was made because fecundity is a numeric quantity that can be reduced additively whereas resistance is a reduction in the probability of infection.

We initially assume that $q_{i}$ can evolve independently in both S and R hosts, and there is no recombination. In the context of adaptive dynamics, this means that mutations in q are introduced in both the S and R genotypes and allele frequencies are allowed to equilibrate in both hosts without recombination. Note that frequencies of S, R and the level of general resistance can affect the prevalence of disease, which can in turn feedback to drive selection. Our approach assumes clonal reproduction and allows associations to evolve between general and specific resistance without recombination—that is, general resistance acts as a completely linked ‘modifier locus’ in which mutations have small cumulative effects. In follow-up simulations, we added an explicit treatment of recombination (see below).

### 2.4 ∣ The model

Our model consists of a system of differential equations, representing the abundance of uninfected hosts that vary in resistance levels and specificities (Equations 1 and 2), hosts infected by the endemic pathogen (Equation 3) and hosts infected by the foreign pathogen (Equation 4). Note that S and R in Equations 1 and 2 represent vectors of general resistance strengths in S and R hosts, where the length n is determined by the number of different general resistance values, $q_{i}$, i∈0…n. Note that different values of $q_{i}$, can evolve in Equations 1 and 2.


(1) $$\frac{dS_{i}}{dt}=S_{i}\left(b-c_{q_{{}_{i}}}-\mu -\gamma N-\frac{1-q_{i}}{N}(\beta_{e}I_{e}+\beta_{f}I_{f})\right)$$

(2) $$\frac{dR_{i}}{dt}=R_{i}\left(b-c_{q_{{}_{i}}}-c_{r}-\mu -\gamma N-\frac{1-q_{i}}{N}\left((1-r)\beta_{e}I_{e}+\beta_{f}I_{f}\right)\right)$$

(3) $$\frac{dI_{e}}{dt}=I_{e}\left(\sum_{i}\frac{\beta_{e}(1-q_{i})\left(S_{i}+(1-r)R_{i}\right)}{N}-\mu \right)$$

(4) $$\frac{dI_{f}}{dt}=I_{f}\left(\sum_{i}\frac{\beta_{f}(1-q_{i})\left(S_{i}+R_{i}\right)}{N}-\mu \right)$$

The demographics for hosts are determined by the mortality rate, μ, the coefficient of density-dependent growth γ and birthrate b. Since infected individuals ($I_{e}$ and $I_{f}$) are sterile and they have no birthrate term. We assume that there is no recovery and that the death rate μ is the same for all individuals, that is, the infection does not induce additional mortality (parameters are summarized in Table 1). The density-dependent transmission was modelled by removing the $\frac{1}{N}$ term from each equation (see Data S1).

### 2.5 ∣ Simulations

General resistance ($q_{i}$) takes values between 0 and 1, in increments of 0.01. For each iteration, we used forward Euler approximations, a common method to generate numerical solutions to dynamical systems (Atkinson, 1991), to run the dynamical system until t=1000, to allow sufficient time for the allele frequencies to converge to equilibrium. After this, we then introduced mutation by taking 5% of the equilibrium genotypes and transferring it to adjacent values of q for both S and R. This algorithm is based on numerically simulating nearly faithful reproduction, as outlined by Metz et al. (1995). In total, this process of mutation and equilibration was iterated up to 150 times, depending on the simulation. This allowed q to reach equilibrium in both S and R. To determine whether our simulations reached a stable equilibrium in terms of the infected and uninfected host proportions, we analysed the last ten evolutionary iterations. If the variation in S, R, $I_{f}$ and $I_{e}$ was below 0.001, the end state was considered stable, otherwise it did not approach equilibrium, and was thus deemed cyclical. All simulations were performed using Python 3 on a Lenovo ThinkPad X1 Yoga Gen 6 laptop.

### 2.6 ∣ Evolution with an endemic pathogen

To establish a baseline for understanding resistance feedback, we first ran models with either specific or general resistance but not both, and a single endemic pathogen. For specific resistance, we varied the cost ($c_{r}$) between 0 and 0.5, and strength of specific resistance (r) between 0 and 1, fixing q=0. For general resistance, we set R=0 and assumed accelerating costs of general resistance (θ=0.5) since prior work has demonstrated that these conditions lead to a single stable evolutionary attractor for general resistance (Boots & Haraguchi, 1999, see Data S3, Figure S4 for rational for accelerating costs). Other parameters were b=1.5, μ=0.2, γ=0.01 and $\beta_{e}=1$. In all cases, starting conditions were a single S and $I_{e}$ individual (we found no dependence on starting abundances).

Next, we asked how the introduction of a specific resistance allele affects the evolution of general resistance, in a population with only a single endemic pathogen and how this would affect the population's vulnerability to foreign pathogens. We allowed the susceptible genotype to reach optimal general resistance (typically 50 mutation iterations), and then introduced the specific resistance gene at low frequency (R=1). Simulations were run for an additional 50 mutation iterations. To determine whether the order of resistance invasion matters, we ran a second set of simulations where specific resistance was allowed to reach equilibrium before general resistance evolution was allowed. For all simulations, we used parameters μ=0.2, γ=0.01, $\beta_{e}=1$ and θ=0.5 and began with S=1 and $I_{e}=1$, with the initial density of R set to R=1 upon its introduction. For all simulations, we recorded the frequency of R, the strength of q, and the prevalence of the endemic pathogen.

We then examined the probability of invasion of a foreign pathogen. For a frequency-dependent model, the growth rate of a pathogen with no recovery and no mortality-virulence is defined in Equation 5.


(5) $$R_{0}=\frac{\beta }{\mu }$$

Therefore, for the foreign pathogen to be able to spread, we must have $R_{0}>1$, which is equivalent to the condition β>μ. For the foreign pathogen, all resistance is given by general resistance, meaning $\beta =\beta_{f}(1-q)$. Therefore, the necessary condition for the spread of the foreign pathogen is $\beta_{f}(1-q)>\mu$. From this, we examined the change in growth rate for a hypothetical foreign pathogen before and after specific resistance is introduced into the simulation.

### 2.7 ∣ Evolution with an endemic and foreign pathogen

To determine how the evolution of general and specific resistances facilitate, not just the initial invasion, but also the establishment of foreign pathogens, we ran a second set of simulations that included a foreign pathogen. The foreign pathogen was introduced at low frequency ($I_{f}=1$) after stable evolutionary equilibrium with the endemic pathogen was reached (100 mutation iterations). We then ran the simulation for another 50 mutation iterations so that general resistance could equilibrate (150 iterations in total). While our model does not allow co-infection, pathogens must compete to infect a limited number of susceptible hosts. We initially assumed an intermediate foreign pathogen transmission rate ($\beta_{f}=0.6$), and as before, we examined evolutionary outcomes across a range of specific resistance strengths and costs following the introduction of the foreign pathogen. In follow-up simulations (see ‘the effect of foreign pathogen transmission rate’ in results), we fixed r=0.8 and varied $\beta_{f}$ from 0 to 1. We used the same adaptive dynamics procedure as the endemic and foreign pathogen model, keeping all other parameters the same as in the single pathogen simulations (μ=0.2, γ=0.01, $\beta_{e}=1$ and θ=0.5).

### 2.8 ∣ The effect of recombination

Our simulations with two pathogens showed the evolution of linkage disequilibrium between the two loci, with general resistance evolving to a stable intermediate strength only in specifically susceptible hosts (see results). To test whether this association would be maintained in the face of recombination, we simplified the model to assume that variation in general resistance was represented by only two alleles of $q:\;Q^{+}={q_{i}}^{*}$, the equilibrium evolved strength in the S hosts (typically q>0, see results) and $Q^{-}={q_{j}}^{*}$, the equilibrium evolved strength in the R hosts (q=0, see results). This led to a total of four genotypes: $Q^{+}R$ (having both general and specific resistance), $Q^{+}S$ (general resistance only), $Q^{-}R$ (specific resistance with no general resistance) and $Q^{-}S$ (double susceptible). We then allowed recombination to occur between the R and Q loci at rate p. This was facilitated through a mating matrix, which determined the baseline number of births $B_{i}$ of each genotype (see Data S4). We used the same cost structure as in our adaptive dynamics model, where the costs of general and specific resistance are additive and applied these as modifiers after calculating the number of new births for each genotype. We used equilibrium abundances from our adaptive dynamics simulations for our initial conditions. First, we confirmed the equilibrium conditions were the same in both our adaptive dynamics simulation and our recombination model when recombination is set to 0 (p=0). We then tested the simulation outcomes when we introduced different levels of recombination (p=0, p=0.005 and p=0.05).

## 3 ∣ RESULTS

### 3.1 ∣ Evolution with an endemic pathogen

**FIGURE 1:**
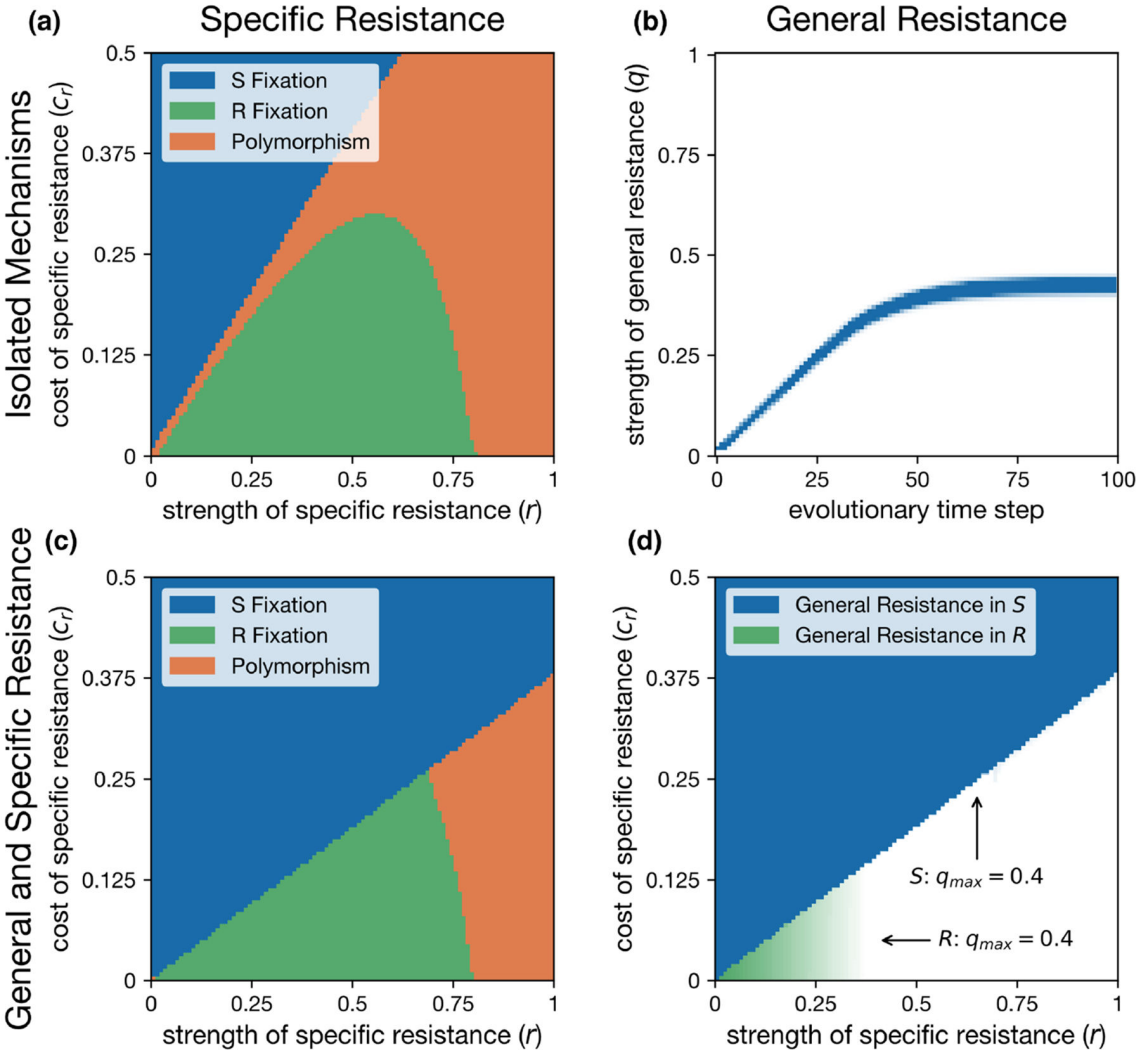
Evolutionary outcomes of specific and general resistance in response to a single endemic pathogen. (a) The fate of a specific resistance gene introduced into a population without general resistance. (b) The evolution of general resistance in the absence of specific resistance (q=0.41, θ=0.5). (c) The fate of a specific resistance gene introduced into a population where general resistance had evolved to equilibrium prior. (d) Strength of evolved general resistance (q) in both S (blue) and R (green) hosts, lighter colours signify lower values of q. Arrows indicate the maximum value of general resistance for either S or R. Other parameters: μ=0.2, γ=0.01, $\beta_{e}=1$ and θ=0.5.

The model including specific resistance but omitting general resistance (i.e., fixing q=0) yielded results consistent with the Antonovics and Thrall (1994) model of specific resistance (Figure 1a). Specific resistance reached fixation at low strength of specific resistance (r) and low resistance costs ($c_{r}$) yet maintained S∕R polymorphism with stronger resistance and greater costs. When specific resistance was not present, general resistance evolved to a stable strength of q=0.41 (Figure 1b).

When both specific and general resistances were allowed to evolve in the same population, the two forms of resistance were highly exclusionary, that is, they rarely cooccur in the same host. Additionally, nearly all parameters that allowed the maintenance of general resistance excluded specific resistance, and vice versa. Relative to our model without general resistance, we found the specific resistance genotype, R, could invade over a smaller region of parameter space when general resistance was present, along with an expansion of the parameter space where S reaches fixation (compare Figure 1a and Figure 1c). However, R was still able to invade and spread in a population with general resistance under a substantial range of parameters, and subsequently drove the loss of general resistance in most cases (white space, Figure 1d). Indeed, general resistance only persisted when specific resistance did not invade (blue region, Figure 1d) or where specific resistance was fixed but at a low strength (light green regions, Figure 1d). Importantly, fixation of specific resistance was not necessary to drive the loss of general resistance. General resistance was lost in all cases where the specific resistance gene evolved to a stable S∕R polymorphism (compare orange area Figure 1c to the white area Figure 1d). Evolutionary outcomes were similar for a model with density-dependent transmission (Figure S1).

The loss of general resistance after the invasion of specific resistance reduced the invasion threshold for a foreign pathogen (Equation 5). General resistance was lost in most regions where the specific resistance, R, was able to invade (Figure 1d), and this resulted in q=0 and a transmission coefficient threshold for foreign pathogen invasion of $\beta_{f}=0.2$. That threshold in the absence of general resistance is lower than in cases where specific resistance failed to invade and the evolved strength of general resistance was q=0.41, translating to a threshold for foreign pathogen invasion of $\beta_{f}=0.339$. In this sense, specific resistance can directly increase the likelihood of invasion by a foreign pathogen by reducing the area of parameter space permitting general resistance.

The order of introduction of the two forms of resistance affected the evolutionary outcomes. Where specific resistance evolved to a stable equilibrium before general resistance was introduced, specific resistance more frequently prevented the invasion and subsequent evolution of general resistance across a broader range of parameters than when general resistance evolves first (see Data S5, Figure S5b). This order of resistance introduction also resulted in the evolution of general resistance in the S hosts for a small subset of the regions with a stable S and R polymorphism, which did not occur when specific resistance was introduced into an established general resistance (Figure 1d and Figure S5b). However, general and specific resistance still maintained a negative association across all parameters except when specific resistance was nearly ineffective (Figure S5b).

### 3.2 ∣ Evolution with an endemic and foreign pathogen

**FIGURE 2:**
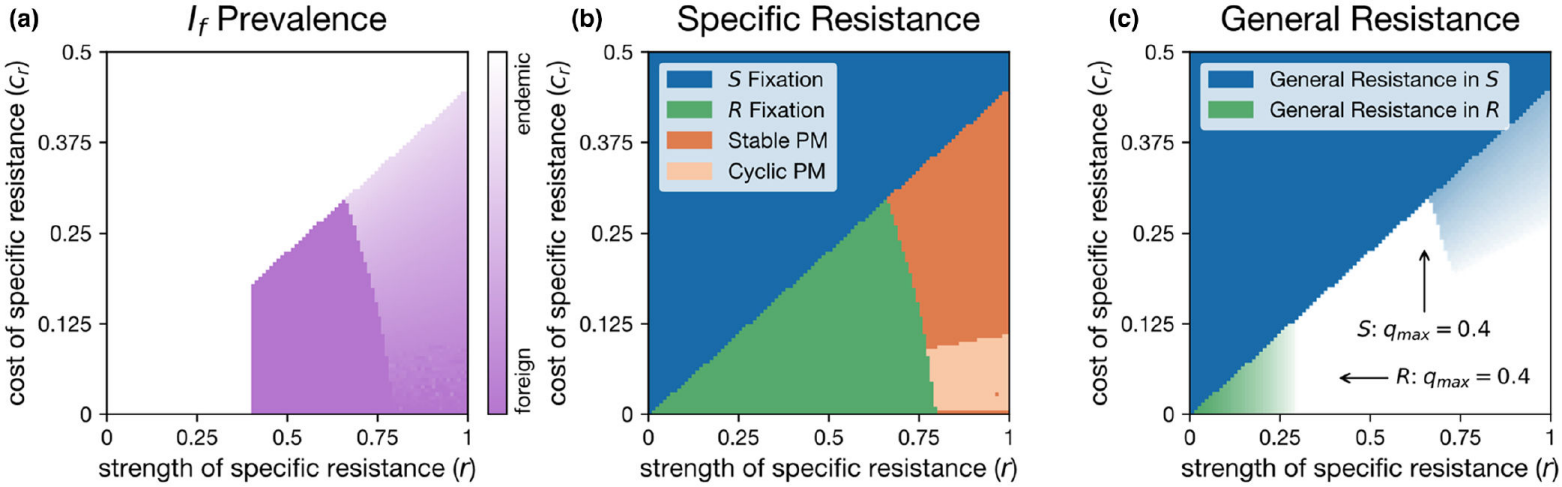
Evolutionary outcomes of specific and general resistance with both the endemic and foreign pathogens. (a) The proportion of hosts infected by the foreign pathogen $I_{f}$ relative to the total infected population. Here, lighter colours signify a lower proportion of $I_{f}$. (b) The fate of a specific resistance gene after the introduction of $I_{f}$. Here, PM signifies S∕R polymorphism. (c): Strength of evolved general resistance (q) in both S (blue) and R (green) genotypes. Lighter colours signify a lower value of quantitative resistance, q. White regions represent parameters where neither S or R maintain any general resistance. Arrows indicate the maximum value of general resistance for either S or R. Other parameters: μ=0.2, γ=0.01, $\beta_{e}=1$, $\beta_{f}=0.6$ and θ=0.5.

The ability of the foreign pathogen to invade and persist was affected by the frequency of the specific resistance genotype, R. Because $\beta_{e}>\beta_{f}$, the endemic pathogen was always more infectious than the foreign pathogen for the S genotype. However, because specific resistance does not affect the foreign pathogen, a necessary condition for the persistence of the foreign pathogen is $\beta_{f}>(1-r)\beta_{e}$. Furthermore, the foreign pathogen failed to persist when R was lost. As a result, in our simulations where $\beta_{f}=0.6$, the foreign pathogen could only invade when r>0.4 (Figure 2a). Indeed, R reached fixation in regions near r=0.4, allowing the foreign pathogen to displace the endemic pathogen (Figure 2a,b). In contrast, parameters that led to stable S and R polymorphisms, led to the persistence of both pathogens, as the endemic pathogen represented by $I_{e}$ is more infectious against S.

**FIGURE 3:**
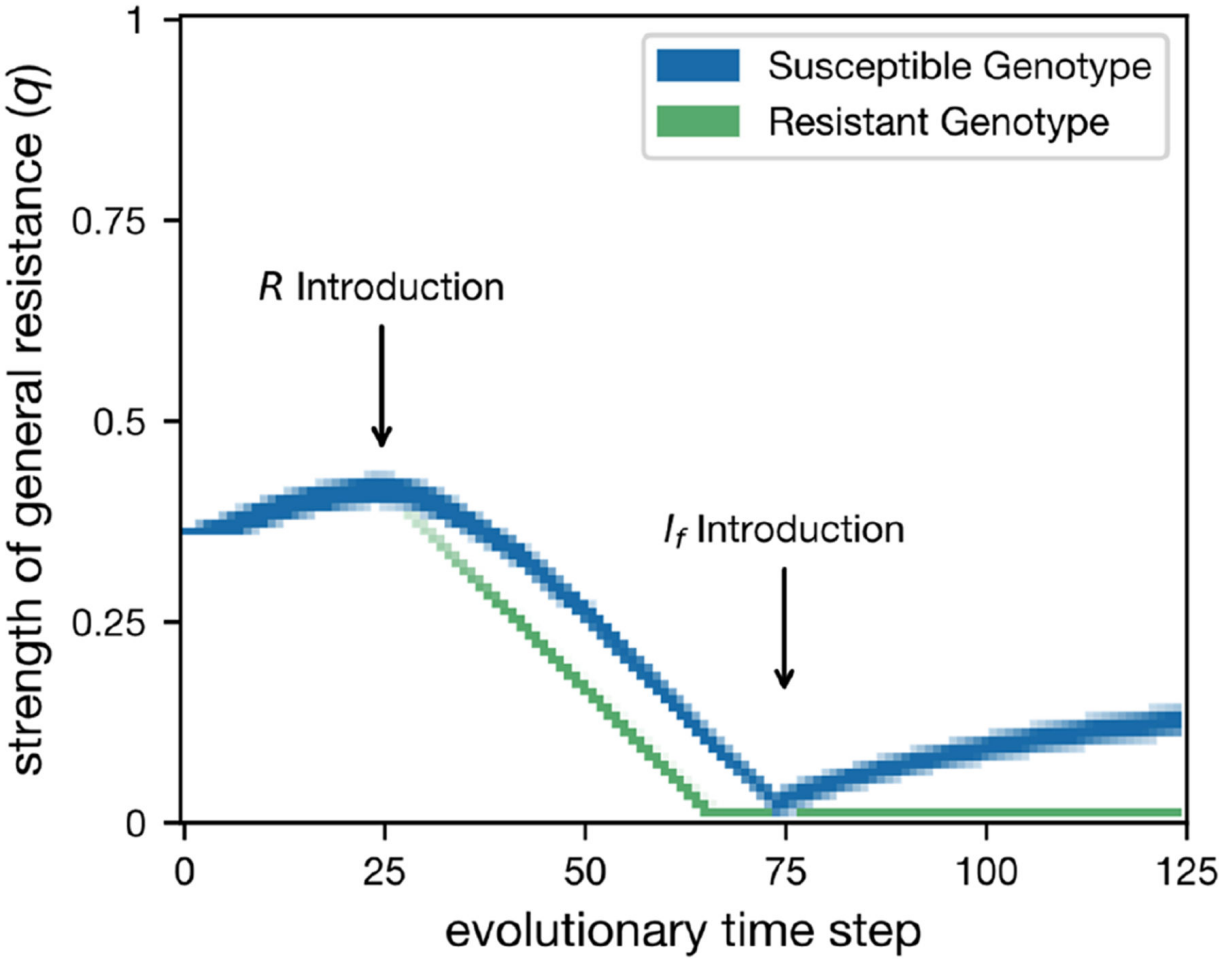
Example simulation showing changes in the strength of general resistance over evolutionary time. The parameters used here result in S∕R polymorphism with S gaining intermediate general resistance. Here, the time course of the simulation has been modified to enhance visual clarity (R introduction at t=25, $I_{f}$ introduction at t=75). Other parameters used: r=0.85, $c_{r}=0.325$, μ=0.2, γ=0.01, $\beta_{e}=1$, $\beta_{f}=0.6$ and θ=0.5.

The introduction of the foreign pathogen also changed the stable frequencies of the S and R genotypes. As in the single pathogen simulations, general and specific resistance still maintained a strong negative association, although not to the same extent (Figure 2c). Relative to the single pathogen simulations (Figure 1c), the regions of S∕R polymorphism and R fixation were slightly expanded (Figure 2b). Unlike the single pathogen simulations, the two pathogen simulations yielded a region of parameter space maintaining both a S∕R polymorphism and positive values for general resistance (q) in S hosts (light blue region near the top right corner of Figure 2c corresponding to the orange region of S∕R polymorphism in Figure 2b). Here, general resistance evolved to an intermediate strength in the S hosts but did not evolve to a positive value in the R hosts (Figure 3). As with the single pathogen simulations, R only gained general resistance when the strength of specific resistance was very weak (green region in the bottom left of Figure 2c). Therefore, S and R polymorphism is maintained with linkage disequilibrium between positive general resistance and the susceptible, S, allele of the specific resistance trait. This region of polymorphism is expanded if the foreign pathogen is more infectious (see ‘The effect of the foreign pathogen transmission rate’ below). In addition, we also ran simulations where all host and pathogen genotypes are present without general resistance prior to allowing general resistance to evolve (Data S5). This resulted in a reduction of the region of R fixation, and an increase in the region of S∕R polymorphism, although we still maintained general resistance in only the S hosts (Figure S6). For density-dependent growth, we saw similar outcomes, with an expanded region of S fixation (Figure S2).

### 3.3 ∣ The effect of the foreign pathogen transmission rate

**FIGURE 4:**
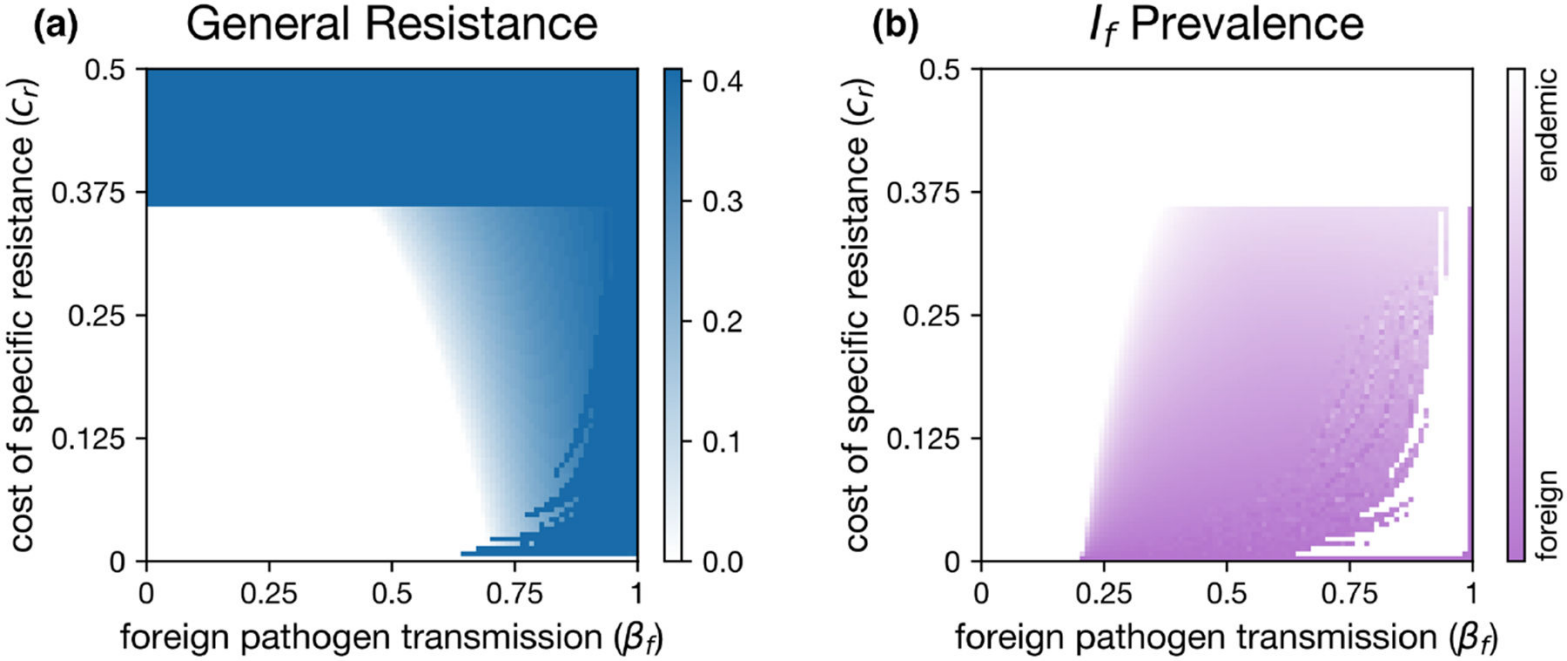
Simulation outcomes for the two genotype, two pathogen model. But vary the foreign pathogen transmission ($\beta_{f}$) instead of the strength of specific resistance (r). After the system reaches equilibrium, we then introduce the foreign pathogen at t=100 and allow the simulation to run for another 50 mutation iterations. (a) Equilibrium values of general resistance for the susceptible genotype, the bar to the right of the plot indicates which colour corresponds to which q value. (b) Equilibrium proportions of pathogen species. The bar to the right of the plot indicates which colour corresponds to which pathogen. For all simulations, μ=0.2, γ=0.01, $\beta_{e}=1$, r=0.8 and θ=0.5.

To determine how the transmissibility of foreign pathogens affects resistance dynamics, we ran simulations varying the foreign pathogen transmission rate ($\beta_{f}$) and costs of specific resistance ($c_{r}$). We focused on regions where general and specific resistances were maintained in polymorphism with the endemic pathogen (r=0.8). Varying the level of foreign pathogen transmission ($\beta_{f}$) had significant effects on the dynamics of a general resistance, notably on the degree of exclusivity between general and specific resistance (Figure 4a). General resistance evolved in the S genotype when the foreign pathogen transmission rate was high, or the cost of specific resistance was high. However, the R genotype never evolved general resistance (q=0), generating a linkage disequilibrium between loci where S often gained intermediate general resistance (light blue region, Figure 4a) while R always maintained q=0. The foreign pathogen was only able to invade and establish at intermediate transmission rates (Figure 4b). At low levels of foreign pathogen transmission (c.a. $\beta_{f}\leq 0.2$) the foreign pathogen's death rate is lower than its highest potential transmission, and thus it cannot invade. At high levels of transmission, its exclusion can be explained by the loss of the specific resistance gene.

### 3.4 ∣ The effect of recombination

**FIGURE 5:**
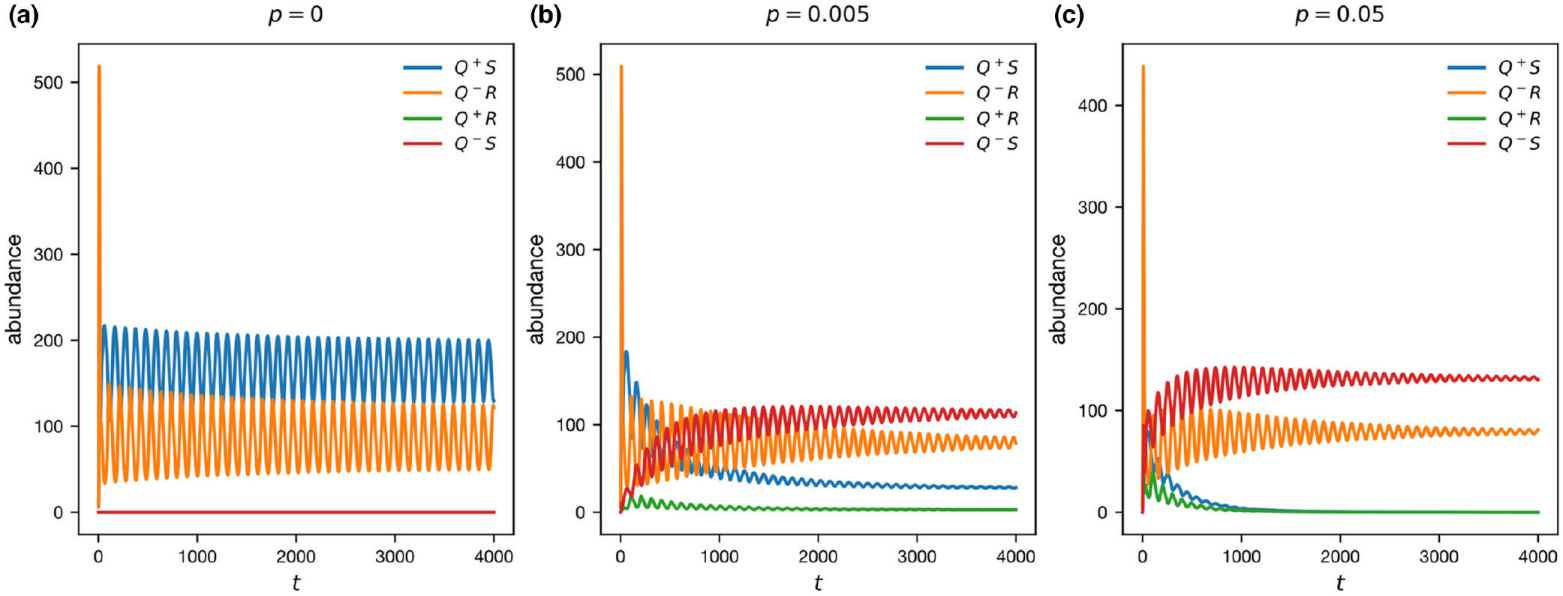
Models incorporating recombination, demonstrating how even low levels of recombination are sufficient to change which genotypes can persist. (a) No recombination. (b) With very low recombination (p=0.005), we see similar results to our adaptive dynamics model, though recombination allows the double susceptible genotype to persist. (c) When we incorporate more recombination (p=0.05) the stable genotypes change to $Q^{-}R$ and $Q^{+}R$. Here, we used parameters taken from the stable equilibrium obtained in adaptive dynamics μ=0.2, γ=0.001, r=0.2, $c_{r}=0.1$, $c_{q}=0.0465$, q=0.091 and $\beta_{f}=0.7$.

We used our recombination model (See Methods and Data S4) to determine whether the observed linkage disequilibrium between general resistance and $S(Q^{+}S)$ and loss of general resistance and $R(Q^{-}R$ was robust to recombination. Without recombination, our results mirrored the results of our adaptive dynamics model and both $Q^{+}S$ and $Q^{-}R$ were maintained in a cyclic polymorphism (Figure 5a). At higher levels of recombination, we saw the complete loss of the $Q^{+}$ allele (Figure 5c). With recombination, the low-cost double susceptible genotype ($Q^{-}S$) is introduced into the population, and rapidly becomes the dominant genotype (red line, Figure 5b,c). This can occur because the presence of the R allele keeps the endemic pathogen at a low prevalence, reducing selection for additional general resistance. Additionally, the frequency of the $Q^{+}$ allele is reduced, such that recombination is an increasingly large fitness cost for $Q^{+}S$, since some of their offspring will recombine to form the cost-ineffective $Q^{+}R$. At sufficiently high levels of recombination, this process leads to the complete loss of $Q^{+}$ (Figure 5c).

## 4 ∣ DISCUSSION

While specific resistance polymorphisms are well documented in natural plant populations (Burdon et al., 1983; Karasov et al., 2014; Laine, 2004), the effects of these polymorphisms on the evolution of general resistance, and the consequences for host-shifts are less clear. In our simulations, with a single, endemic pathogen, we found that the evolution of specific resistance against the endemic pathogen can reduce the pathogen pressure enough to render general resistance non-cost effective, even when the specific resistance gene remains polymorphic. This leaves populations more susceptible to novel pathogen invasion as all hosts lose general, broad-spectrum resistance. Moreover, in our simulations with both endemic and foreign pathogens, the evolution of stable polymorphisms for specific resistance also facilitated the persistence of foreign pathogens by creating an ecological niche for foreign pathogen establishment.

With a single endemic pathogen, our model shows that general and specific resistance evolve in a highly exclusionary fashion. A similar result was found by Frank (2000), who used a Lotka-Volterra approach with explicit modelling of parasite numbers to show a negative correlation between unlinked genes for general and specific resistance. A key result from our model is that the exclusion of general resistance occurs even if specific resistance does not reach fixation but remains polymorphic with susceptible genotypes. Indeed, we find that conditions that lead to a stable polymorphism in specific resistance always drive the complete loss of general resistance. In contrast, when specific resistance cannot invade, hosts often maintain intermediate strengths of general resistance (although using decelerating costs in this scenario would lead to general resistance polymorphisms).

Critically, the loss of general resistance lowers the invasion threshold for foreign pathogens, leaving host populations more vulnerable to invasion by the foreign pathogen. This finding complements the results by Lerner et al. (2021) who showed that host resistance structure can affect probabilities of foreign pathogen infection. The Lerner et al. model assumed a single locus that had pleiotropic effects on resistance to endemic and foreign pathogens, which often led to an increased invasion probability by a foreign pathogen. However, this model did not propose a mechanism for how pleiotropy between resistance to the endemic and foreign pathogen could emerge, whereas our model yields a strong negative association between specific and general resistance de novo. In our model, this linkage disequilibrium was always present with only the endemic pathogen (Figure 1), many parameters still led to a complete disassociation even with both pathogens (Figure 2). In their study, Lerner et al. (2021) a significant positive correlation between the resistance of 28 half-sib families of *S. vulgaris* to its endemic anther smut pathogen and to a foreign anther smut pathogen, consistent with a variable general resistance. However, they also found several significant outliers, which were highly susceptible to the foreign pathogen yet resistant to the endemic pathogen. This result cannot be easily explained by pleiotropy but is consistent with specifically resistant genotypes reducing investment into general resistance. Importantly, this means that host resistance structure can likely favour foreign pathogen invasion, even if there is no direct negative pleiotropy between resistance to endemic and foreign pathogens. Furthermore, given certain parameters (low cost to specific resistance and relatively low foreign pathogen transmission rates), the introduction of the foreign pathogen does not significantly alter the resistance structure of the host population, thus maintaining the heightened susceptibility to additional foreign pathogen introductions.

The ability of the foreign pathogen to not only invade but also persist given the presence of the endemic pathogen was determined by the host resistance structure. The success of the foreign pathogen was ultimately tied to the frequency of specific resistance, as the foreign pathogen was more infectious against the resistant genotype, which lacked general resistance. Parameters conferring strong specific resistance to the endemic pathogen and low to moderate costs lead to specific resistance polymorphisms that supported the coexistence of both pathogens. Pathogen coexistence in turn had important feedback on resistance structure. For example, when foreign pathogen transmission and the cost of specific resistance are intermediate, both general resistance and polymorphism for specific resistance were maintained. This outcome was not possible with a single pathogen species.

It is important to note that the maintenance of both types of resistance occurred through an emergent linkage disequilibrium between general resistance and the alternate alleles for specific resistance, where genotypes were favoured that had one form of resistance but not both. General resistance only evolved in hosts that lacked the allele for specific resistance. However, this association was broken by even modest levels of recombination, which led to the loss of general resistance for all hosts. With regard to recombination, our model differs from the results of Frank (2000). In that model, Frank (2000) found that recombination reduced the negative correlation between general and specific resistance, but the association was not broken unless hosts were freely recombining. In vivo, general resistance is likely controlled by many loci, unlike our recombination implementation (Corwin & Kliebenstein, 2017). If multiple general resistance loci are considered, then recombination would mean more genotypes with an intermediate general resistance. This could increase the fitness of $Q^{+}S$ genotypes, potentially allowing for the stable coexistence of general and specific resistance with recombination. Still, the explicit genetic results demonstrate a high degree of sensitivity to recombination; the equilibrium state likely depends greatly on the degree of linkage.

In natural settings, novel pathogen invasions are a frequent occurrence (Parker & Gilbert, 2004). While much of the focus on the risk of host-shifts has been on the phylogenetic distance between hosts (Parker et al., 2015; Streicker et al., 2010) and between pathogens (Longdon et al., 2014), host investment in general versus specific resistance could also be a critically important factor. We found the counterintuitive result that foreign pathogens with intermediate transmission coefficients were best able to invade and persist. Foreign pathogen frequency is maximized when its transmission rate is not high enough to eliminate specific resistance so that it does not lose hosts with specific resistance that are its most opportune host. If left with just hosts carrying susceptibility at the specific resistance trait, the foreign pathogen will eventually be excluded by the endemic pathogen's superior transmission potential. The lower transmission rate of a foreign pathogen relative to an endemic pathogen is often due to being maladapted to parasitizing their new hosts (Antonovics et al., 2002; Longdon et al., 2014), but in light of our results, some degree of maladaptation may actually help a novel pathogen become established within a new host population where it avoids eliminating variation for resistance specific to the endemic pathogen. The optimality of intermediate transmission rates has implications for the natural history of successful pathogen spillovers. Previous research has shown a clear link between a foreign pathogens transmission rate and the phylogenetic distance between ancestral and novel hosts (Kamath et al., 2016). In light of our model, this would imply that a host is most vulnerable in the long term to foreign pathogens whose ancestral host is only moderately distant from the novel host.

Our outcomes are highly dependent on the cost structure of general and specific resistance. In our model, selection imposed by an endemic pathogen causes specific resistance with low costs to readily invade and displace costly general resistance, with consequences for invasion by other pathogens. While fitness costs of resistance have been demonstrated in a range of organisms including vertebrates (Armitage et al., 2003; Lochmiller & Deerenberg, 2000), invertebrates (Bartlett et al., 2018; Kraaijeveld et al., 2001) and plants (Biere & Antonovics, 1996; Karasov et al., 2014; Susi & Laine, 2015; Tian et al., 2003), we still have little knowledge of the cost differentials between general and specific resistance. In a rare example, Susi and Laine (2015) quantified the costs of general and specific resistance to powdery mildew in wild populations of *Plantago lanceolata* and found much higher costs associated with a general resistance. In addition to magnitude, the shape of the trade-off surface plays an important role in determining evolutionary outcomes (Boots & Haraguchi, 1999), yet, empirical evidence for whether costs are accelerating, or decelerating is still lacking.

Although our model was based on anther-smut, which is a sterilizing pathogen with the frequency-dependent transmission, our results likely apply more broadly. Beyond anther-smut, this combination of transmission mode and sterility-virulence is characteristic of many sexually transmitted diseases in animals (Lockhart et al., 1996). We also found that changing the transmission mode to mass-action density dependence did not result in major qualitative shifts in outcome (See Data S1, Figures S1 and S1). However, the effects of virulence mode (sterility vs. mortality-inducing) and recovery on the evolutionary dynamics of general and specific resistance remains an open question (Ewald, 1991; Leggett et al., 2017). Indeed, future studies that incorporate mortality-inducing virulence could potentially yield new insights as tradeoffs between virulence and transmission that are likely to evolve, especially during new pathogen emergences (Visher et al., 2021).

The results of our model show that the genetic structure of host populations with respect to variation in two important and common forms of resistance can affect the risk of foreign pathogen invasion and subsequent establishment. We show that host investment in specific resistance at the expense of general resistance is a common evolutionary outcome against a single endemic pathogen, and relaxes conditions required for invasion by foreign pathogens. In the presence of a single endemic pathogen, the evolution of stable polymorphic-specific resistance is enough to drive the complete loss of general resistance. In the presence of both endemic and foreign pathogens, polymorphism for specific resistance can drive the coexistence of both pathogens, and in some cases, maintenance of both general and specific resistance. Taken together our results show that understanding the genetic structure of host resistance, and the evolutionary drivers moulding that structure are important, not just for predicting dynamics with endemic pathogens, but also for the success of cross-species transmission events.

## Supplementary Material

Supporting Information

## Figures and Tables

**TABLE 1:** Summary of parameters used in our model.

Parameter	Model function
b	Birth rate
μ	Host mortality rate
γ	Density-dependent population regulation
$q_{i}$	Strength of general resistance
r	Strength of specific resistance
$c_{r}$	Cost of specific resistance
$c_{q}$	Cost of general resistance, proportional to $q_{i}$
$\beta_{e}$	Transmission rate of endemic pathogen
$\beta_{f}$	Transmission rate of a foreign pathogen
θ	Curvature of general resistance cost function

## Data Availability

The code and scripts used for this paper are available via GitHub at http://github.com/svhulse/qr-model. We have uploaded the repository to zenodo (https://zenodo.org/record/7686242#.Y_535x_MKUk) DOI:10.5281/zenodo.7686242
