# Supplementary material for: Specific resistance prevents the evolution of general resistance and facilitates disease emergence
Source: J Evol Biol. Author manuscript; Available in PMC 2023 Jul 3. (PMC10316961; doi:10.1111/jeb.14170)
Supplement: Supporting Information [file NIHMS1908405-supplement-Supporting_Information.docx]

Supplementary Materials for Specific resistance prevents the evolution of general resistance and facilitates disease emergence

Supplementary Note 1: Density Dependent Transmission

To test the generality of our model, we modified our equations to simulate density dependent transmission. To do so, we modify Eqns. 1-4 from the main text by removing $\frac{1}{N}$ from the transmission terms to yield the following equations.

$$\begin{aligned} \frac{dS_{i}}{dt}=S_{i}\left( b-c_{q_{i}}-\mu-\gamma N-(1-q_{i})\left( \beta_{e}I_{e}+\beta_{f}I_{f} \right) \right) \#(1)\# \end{aligned}$$

$$\begin{aligned} \frac{dR_{i}}{dt}=R_{i}\left( b-c_{q_{i}}-c_{r}-\mu-\gamma N-(1-q_{i})\left( \left( 1-r \right)\beta_{e}I_{e}+\beta_{f}I_{f} \right) \right) \#(2)\# \end{aligned}$$

$$\begin{aligned} \frac{dI_{e}}{dt}=I_{e}\left( \sum_{i} \beta_{e}\left( 1-q_{i} \right)\left( S_{i}+\left( 1-r \right)R_{i} \right)-\mu\right) \#(3)\# \end{aligned}$$

$$\begin{aligned} \frac{dI_{f}}{dt}=I_{f}\left( \sum_{i} \beta_{f}\left( 1-q_{i} \right)\left( S_{i}+R_{i} \right)-\mu\right) \#(4)\# \end{aligned}$$

With this framework, we ran simulations with just the endemic pathogen (Fig. S1) as well as with both the endemic and foreign pathogen (Fig. S2). For these simulations, we maintain all parameters used for simulations in our main test except for the transmission terms ($\beta_{e}=0.01$ and $\beta_{f}=0.0085$), which must be modified to account for the different units of transmission. Interestingly, for our density dependent simulation with the endemic and foreign pathogen, we often see the extinction of the resistant genotype when specific resistance is both highly effective and cheap (bottom right corner, Fig. S2B). In these scenarios, when the foreign pathogen is first introduced, the endemic pathogen is at a very low abundance due to the strength of specific resistance. This allows the foreign pathogen to become rapidly dominant, causing the extinction of $R$. From this point, without $R$ present, the endemic pathogen can outcompete the foreign pathogen (Fig. S2A, bottom right corner).


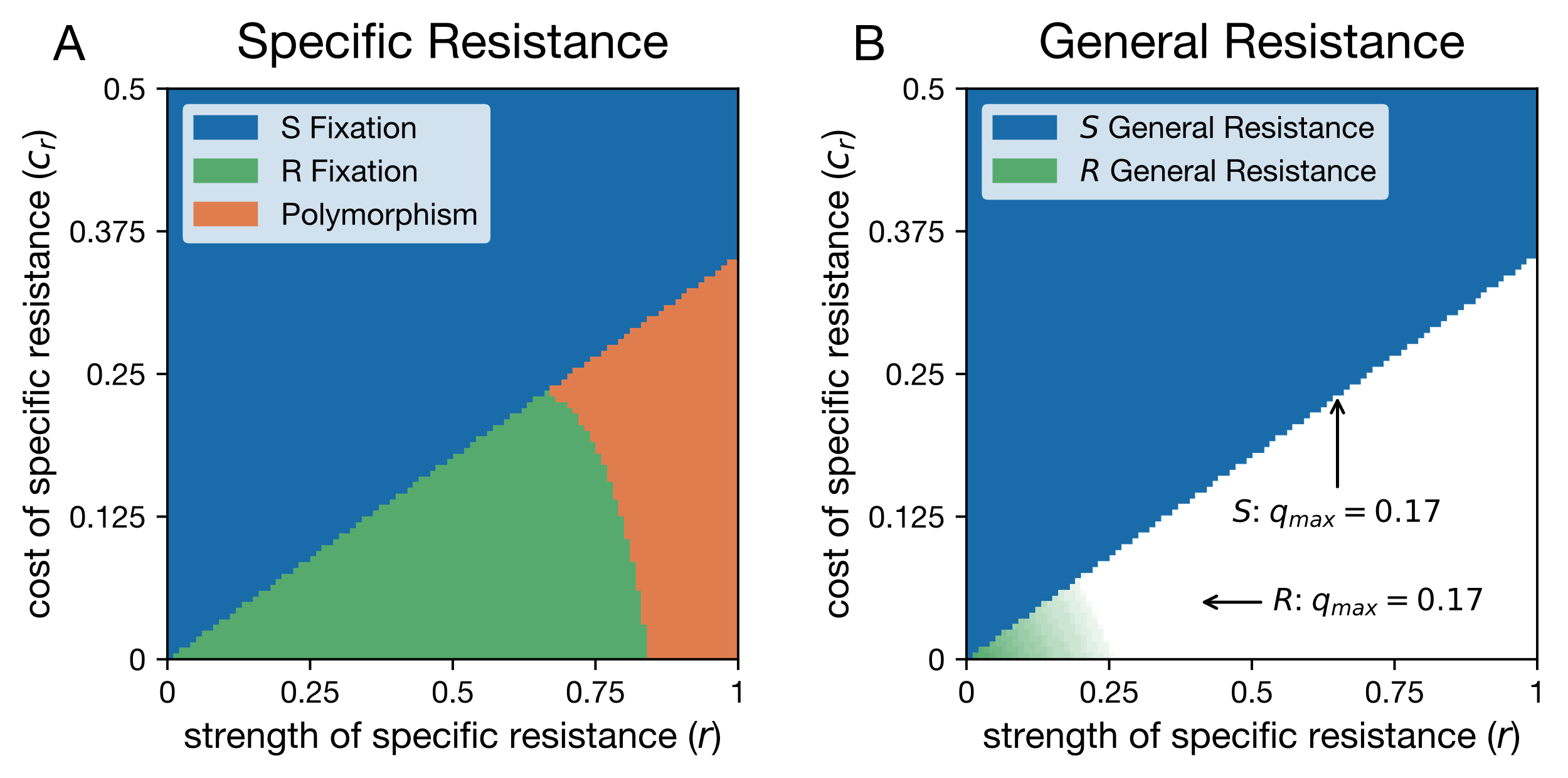


**Figure S1:** Evolutionary outcomes of specific and general resistance in response to a single endemic pathogen with density dependent transmission. (A): The fate of a specific resistance gene which is present in the population where general resistance has evolved to equilibrium prior and disease transmission is density dependent. (B): Strength of evolved general resistance ($q$) in both $S$ (blue) and $R$ (green) hosts, lighter colors signify lower values of $q$. Arrows indicate the maximum value of general resistance for either $S$ or $R$. Other parameters: $\mu=0.2,\gamma=0.01,\beta_{e}=0.01,$ and $\theta= 0.5$.


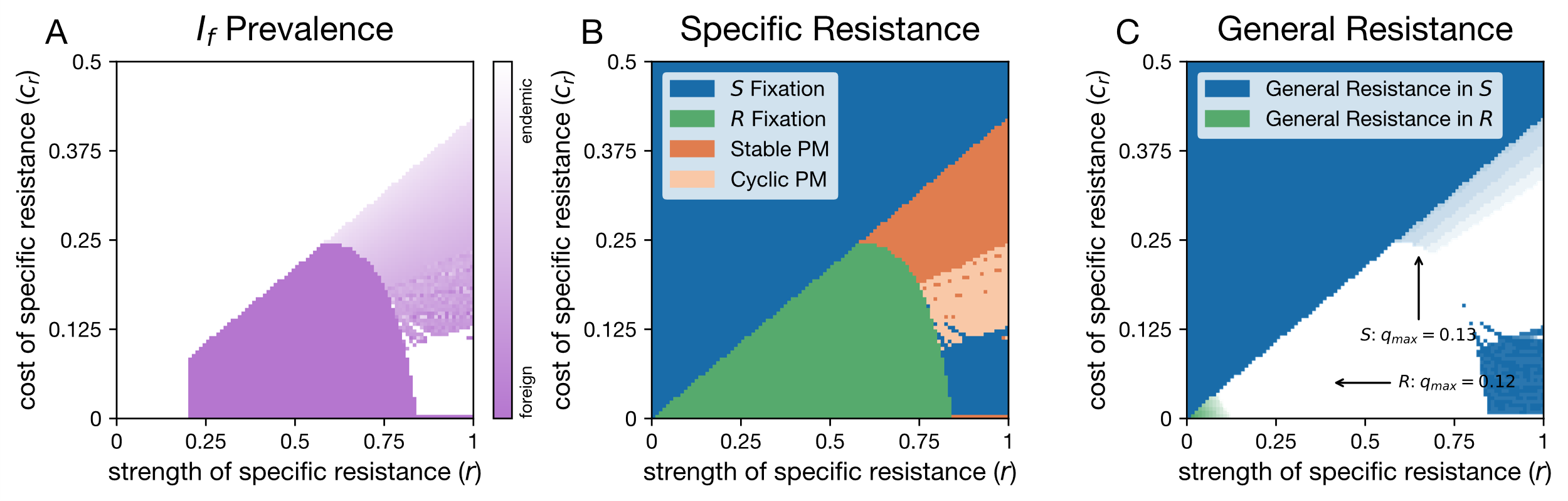


**Figure S2:** Evolutionary outcomes of specific and general resistance with both the endemic and foreign pathogens having density dependent transmission. (A): The proportion of hosts infected by the foreign pathogen $I_{f}$ relative to the total infected population. Here, lighter colors signify a lower proportion of If. Here, PM signifies $S/R$ polymorphism. (C): Strength of evolved general resistance ($q$) in both $S$ (blue) and $R$ (green) genotypes. Lighter colors signify lower value of quantitative resistance, $q$. White regions represent parameters where neither $S$ or $R$ maintain any general resistance. Arrows indicate the maximum value of general resistance in either for either $S$ or $R$. Other parameters: $\mu=0.2$, $\gamma=0.01$, $\beta_{e}=0.01$, $\beta_{f}=0.0085$, and $\theta=0.5$.

Supplementary Note 2: Derivation of Optimal General Resistance

We consider a monomorphic population with a fixed level of qualitative resistance, with only one pathogen present. In this simplified version of our model, the equilibrium density for the host is given by Equation 5, while the equilibrium density of infected individuals is given by Equation 6. Here, we substitute $q$ for $1-q$, treating $q$ as transmission rather than resistance to simplify the equations. The analytical results can then be transformed back into resistance.

$$\begin{aligned} S^{*}=\frac{\mu\left( f\left( q \right)-q \right)}{\gamma q}\#\left( 5 \right) \end{aligned}$$

$$\begin{aligned} I^{*}=\frac{\left( f\left( q \right)-q \right)\left( q-\mu\right)}{\gamma q}\#\left( 6 \right) \end{aligned}$$

Therefore, the equilibrium total population simplifies to

$$\begin{aligned} N^{*}=\frac{f\left( q \right)-q}{\gamma}\#\left( 7 \right) \end{aligned}$$

We can then analyze how quantitative resistance will evolve by taking the derivative of the host fitness with respect to the level of quantitate resistance (Eqn. 8). Here, we assume only a single endemic pathogen, with $\beta_{e}=1$ for simplicity. From here, we can derive the effect of quantitative resistance on host fitness. We can then substitute in the equilibrium values of $S, I, N$ and calculate the optimal value of $q$. With accelerating costs, we see a single CSS (Boots and Haraguchi, 1999), thus the optimum can be calculated by solving for when the derivative of $S$ with respect to $q$ is zero.

$$\begin{aligned} \frac{\partial}{\partial q}\frac{dS}{dt}=S\left( f^{'}\left( q \right)-r\frac{I}{N} \right)\#\left( 8 \right) \end{aligned}$$

To determine the extent to which the evolution of general resistance depends on the strength of specific resistance ($r$). We consider a monomorphic population with a fixed level of specific resistance, and only one pathogen genotype present with $\beta_{e}=1$ for simplicity. For the case of accelerating costs, there is a single optimum level of general resistance which depends both on the strength of specific resistance and background rate of mortality. We can then calculate the rate of growth for a foreign pathogen ($g$) for the population at equilibrium (Eqn. 9).

$$\begin{aligned} \frac{dI_{f}}{dt}=I_{f}\left( \frac{(1-q)\beta_{f}S}{N}-\mu\right)=I_{f}g\#\left( 9 \right) \end{aligned}$$

When only the endemic pathogen is present, increasing the strength of specific resistance ($r$) leads to a reduction in the optimal level of general resistance ($q$; Fig. S3A), and an increased ability for foreign pathogen to invade (Fig. S3B).


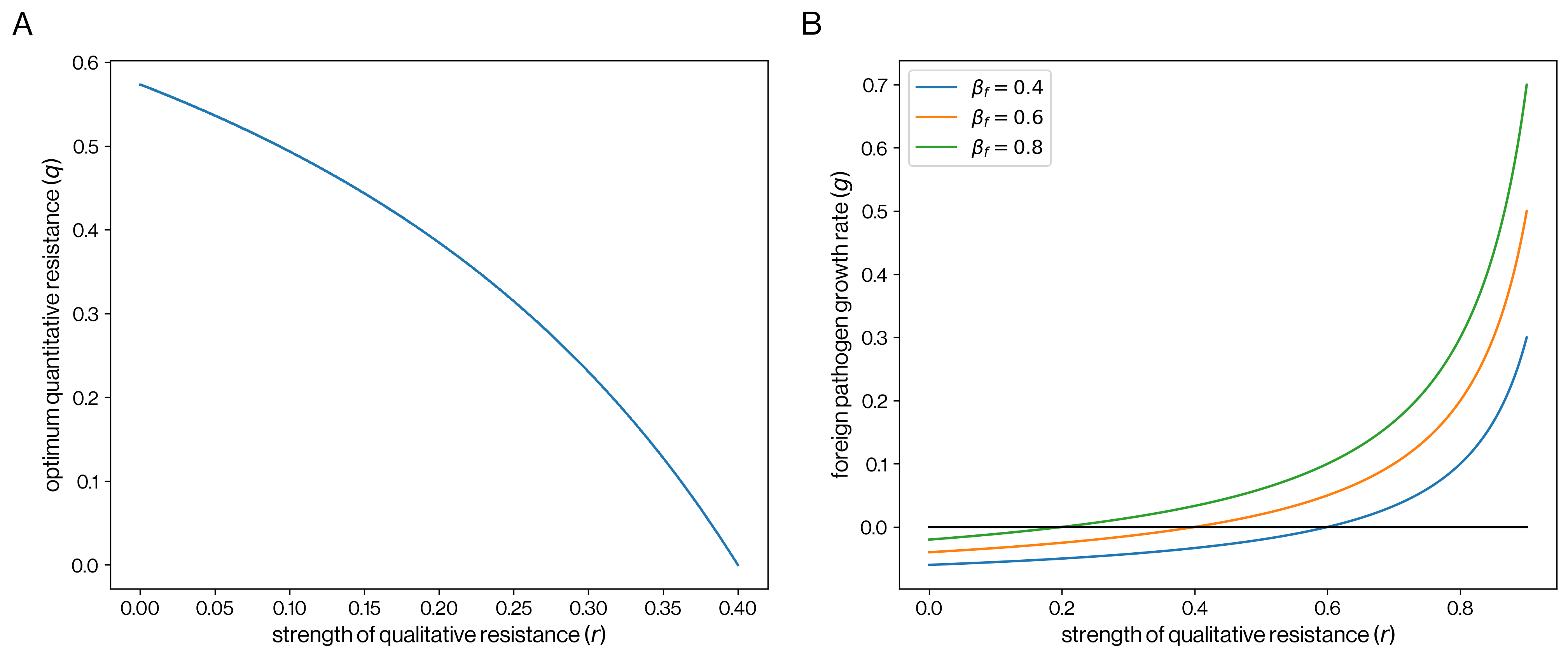


**Figure S3:** (A): The effect background specific resistance on the optimal level of general resistance. (B): The effect of specific resistance on the growth rate of a foreign pathogen with various levels of host susceptibility to foreign pathogens. Here we assume that the level of specific resistance is fixed, and the host population has reached an optimal value for general resistance. Other parameters: $\mu= 0.1, \gamma= 0.01, k = 0.5, t = 0.5$.

Supplementary Note 3: Rational for Accelerating Costs

We assume that a given multi-locus trait has $n$ different alleles, each with independent, additive fitness effects. For each allele, we assume independent, random costs and benefits, given by an exponential distribution with parameter $\lambda= 1$. Then, for each allele, the change in fitness is given by the difference between the costs and benefits. We then assume that mutations will be introduced into the population in order of their fitness impact, with the most beneficial mutations occurring first. With these assumptions, we recapitulate an accelerating cost function (Fig. S4).


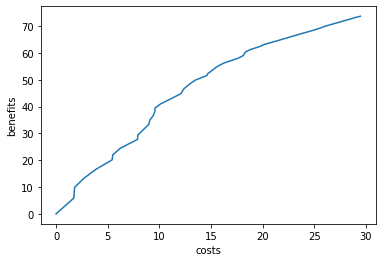


**Figure S4:** Simulation of cost function for a multi-locus trait. Here, we assume that there are $n = 100$ alleles conferring random costs and benefits, with the most optimal traits being introduced first. We assume that traits with greater costs than benefits will not become fixed, thus only beneficial mutations are plotted.

Supplementary Note 4: Mating matrix for testing recombination effects between general and specific resistances.

We assume there are two general resistance alleles, $Q^{+}={q_{i}}^{*}$*,* the equilibrium evolved level in the $S$ background (typically $q>0$, see results), and $Q^{-}={q_{j}}^{*}$*,* the equilibrium evolved level in the $R$ background (typically $q=0$*, see results*). This leads to four host genotypes (Table S1).

**Table S1:** Genotype descriptions for recombination model

| Genotype | Parent | Phenotype |
| --- | --- | --- |
| $H_{1}$ | Q^+^ R | General and specific resistance |
| $H_{2}$ | Q^+^ S | General resistance only |
| $H_{3}$ | Q^-^ R | Specific resistance only |
| $H_{4}$ | Q^-^ S | No resistance |

In a haploid system, we assume that $1-p$ fraction of the births are parental types. The frequencies of each freely recombining allele are described below (Table S2).

**Table S2:** Individual allele frequencies

| Allele | Allele Frequency | Frequency calculation |
| --- | --- | --- |
| Q^+^ | a | $(H_{1}+H_{2})/N_{H}$ |
| Q^-^ | 1-a | $(H_{3}+H_{4}) /N_{H}$ |
| R | b | $(H_{1}+H_{3}) /N_{H}$ |
| S | 1-b | $(H+H_{4}) /N_{H}$ |

Where $N_{H}$ is the total number of healthy hosts and is given as $N_{H}={(H}_{1}+H_{2}+H_{3}+H_{4})$. The adjusted baseline number of births for each genotype can then be expressed as:

$$B_{1}=\left( b-k_{j}-c \right)*\left( H_{1} \left( 1-p \right)+N_{H}pab \right)$$

$$B_{2}=\left( b-k_{j} \right)*\left( H_{2} \left( 1-p \right)+N_{H}pa(1-b) \right)$$

$$B_{3}=\left( b-c \right)*\left( H_{3} \left( 1-p \right)+N_{H}p\left( 1-a \right)b \right)$$

$$B_{4}=b*\left( H_{4} \left( 1-p \right)+N_{H}p(1-a)(1-b) \right)$$

We assumed that costs of resistance were accumulated after the baseline birth of each genotype (e.g. costs accumulated by the offspring, not the maternal parents). This gives the general growth form for each of the four genotypes as:

$$\begin{aligned} \frac{dH_{1}}{dt}=B_{1}-H_{1}\left( \mu+\gamma N+\left( 1-q^{*} \right)\left( {\left( 1-r \right)\beta}_{e}\frac{I_{e}}{N}+\beta_{f}\frac{I_{f}}{N} \right) \right)\# \end{aligned}$$

$$\begin{aligned} \frac{dH_{2}}{dt}=B_{2}-H_{2}\left( \mu+\gamma N+\left( 1-q^{*} \right)\left( \beta_{e}\frac{I_{e}}{N}+\beta_{f}\frac{I_{f}}{N} \right) \right)\# \end{aligned}$$

$$\begin{aligned} \frac{dH_{3}}{dt}=B_{3}-H_{3}\left( \mu+\gamma N+{\left( 1-r \right)\beta}_{e}\frac{I_{e}}{N}+\beta_{f}\frac{I_{f}}{N} \right)\# \end{aligned}$$

$$\begin{aligned} \frac{dH_{4}}{dt}=B_{4}-H_{4}\left( \mu+\gamma N+\beta_{e}\frac{I_{e}}{N}+\beta_{f}\frac{I_{f}}{N} \right)\# \end{aligned}$$

Supplementary Note 5: Introducing Specific Resistance Prior to General

To determine whether the order of resistance introduction effected the ultimate outcome, we ran simulations where we began with both $S$ and $R$ in equal proportion. In these simulations, both genotypes began with no general resistance and were then allowed to evolve to their stable level of $q$. We tested this for both the single, and double pathogen simulations.

For the single pathogen simulations, we used the same parameter values as in the simulation presented in the main text ($b=1.5,\mu=0.2,\gamma=0.01,\beta_{e}=1,\theta=0.5$). We allowed for 50 evolutionary time steps for general resistance to reach equilibrium. Compared to when specific resistance invades a population with equilibrium general resistance, we observed several notable differences. First, we found a small region where $S$ maintained intermediate general resistance in polymorphism with $R$. Additionally, we saw an increase in parameter space in which the resistant genotype can be sustained.

**
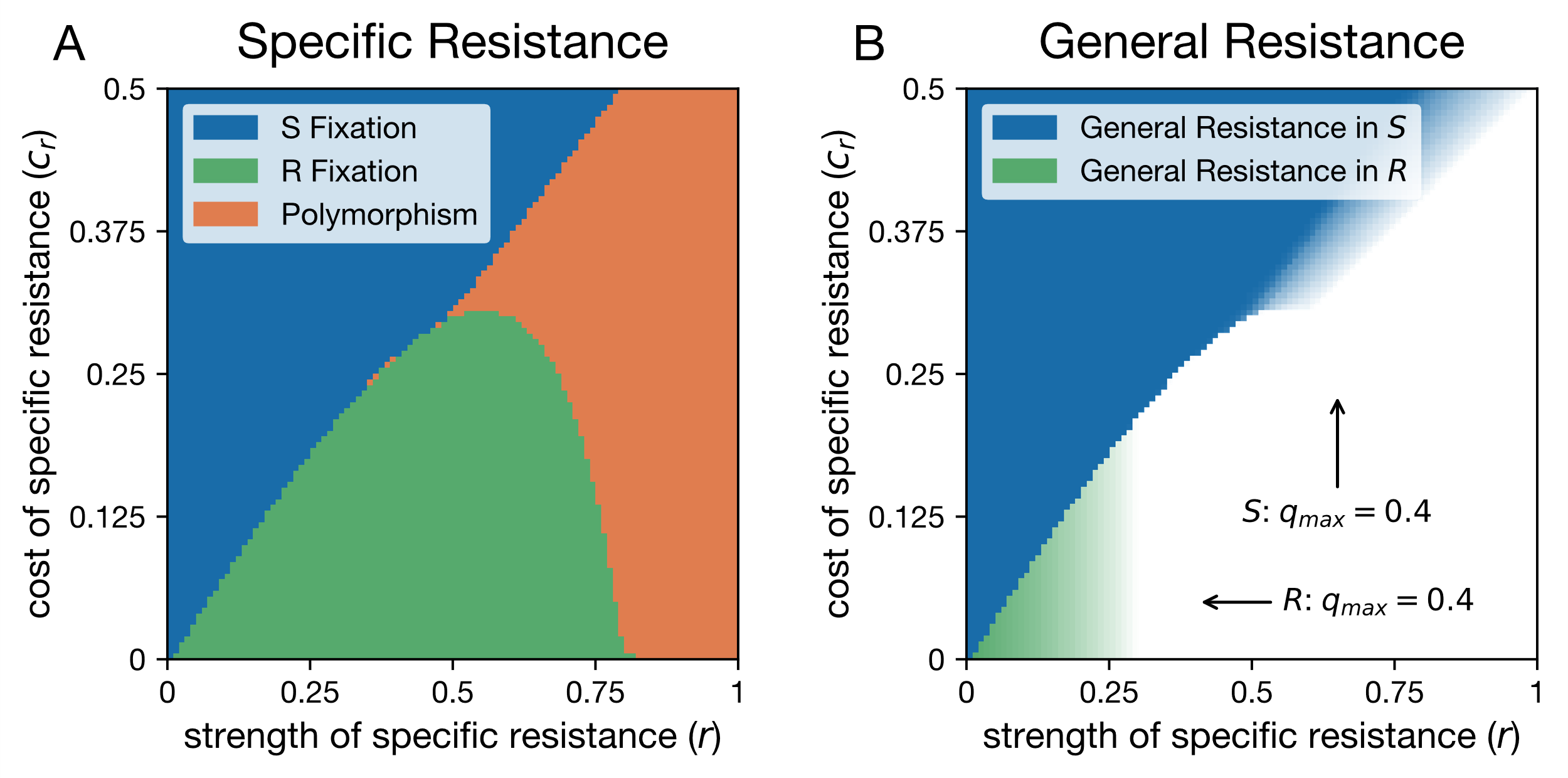
**

**Figure S5:** (A): The fate of a specific resistance gene which is present in the population before general resistance is allowed to evolve. (B): Level of evolved general resistance (q) in both $S$ (blue) and $R$ (green) genotypes, lighter colors signify lower values of $q$. Arrows indicate the maximum value of general resistance in either for either $S$ or $R$. Other parameters: $\mu=0.2$, $\gamma=0.01$, $\beta_{e}=1$, and $\theta=0.5$.

For the two-pathogen system, we began our simulations with $S$ and $R$ present in equal proportions as well as both pathogens present in equal proportions, again beginning with no general resistance. For the foreign pathogen, we assume a transmission rate of 0.5, but maintain all other parameters as before. We then allowed the simulation to run for 50 evolutionary iterations to allow general resistance to equilibrate. Here, we again saw deviations from the outcomes in the general resistance first simulations, corresponding to novel areas where specific resistance could be maintained.


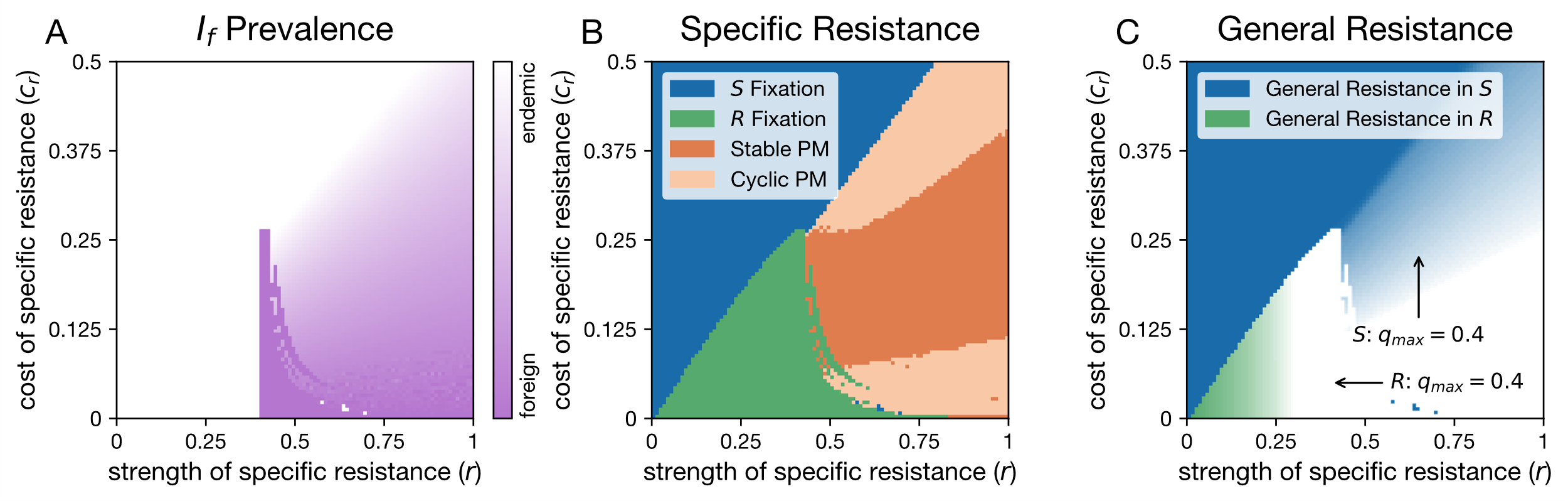


**Figure S6:** Evolutionary outcomes of specific and general resistance with both the endemic and foreign pathogens when specific resistance is present prior to general. (A): The proportion of $I_{f}$ relative to the total infected population. Here, lighter colors signify a lower proportion of $I_{f}$. (B): The fate of a specific resistance gene after the introduction of $I_{f}$. Here, PM signifies $S/R$ polymorphism. (C): Level of evolved general resistance ($q$) in both $S$ (blue) and $R$ (green) genotypes for the two-pathogen model. Lighter colors signify lower value of $q$. Arrows indicate the maximum value of general resistance in either for either $S$ or $R$. Other parameters: $\mu=0.2$, $\gamma=0.01$, $\beta_{e}=1$, $\beta_{f}=0.6$, and $\theta=0.5$.

Supplementary References

Antonovics, J., Thrall, P., 1994. The cost of resistance and the maintenance of genetic polymorphism in host—pathogen systems. Proc. R. Soc. Lond. B 257, 105–110. https://doi.org/10.1098/rspb.1994.0101

Boots, M., Haraguchi, Y., 1999. The Evolution of Costly Resistance in Host‐Parasite Systems. The American Naturalist 153, 359–370. https://doi.org/10.1086/303181
